# Association between serological indicators of past contacts with *Herpesviridae* and a slower resolution of chronic spontaneous urticaria in children

**DOI:** 10.3325/cmj.2023.64.67

**Published:** 2023-04

**Authors:** Anamarija Čavčić, Oktavija Đaković Rode, Vladimir Trkulja

**Affiliations:** 1 Department of Pediatrics, University Hospital Center Zagreb, Zagreb, Croatia; 2 Division of Medical Virology, Department of Microbiology, Dr. Fran Mihaljević University Hospital for Infectious Diseases, Zagreb, Croatia; 3 Department of Pharmacology, University of Zagreb School of Medicine, Zagreb, Croatia

## Abstract

**Aim:**

To evaluate the relationship between serological indicators of *Herpesviridae* infection and evolution of symptoms in children with chronic spontaneous urticaria (CSU).

**Methods:**

In this observational study, consecutive children with CSU underwent, at presentation, clinical and laboratory work-up, autologous serum skin test (ASST) to identify autoimmune urticaria (CAU), disease severity assessment (urticaria activity score 7, UAS7), serological diagnostics for Epstein-Barr virus (EBV), cytomegalovirus (CMV), human herpes virus-6 (HHV-6), and parvovirus B19, as well as for *Mycoplasma pneumoniae* and *Chlamydia pneumoniae*. Children were re-assessed at 1, 6, and 12 months after the commencement of antihistamine/antileukotriene treatment.

**Results:**

None of the 56 included children had an acute CMV/EBV or HHV-6 infection, but 17 (30.3%) had IgG antibodies against CMV, EBV, or HHV-6 (five were also seropositive for parvovirus B19); 24 (42.8%) suffered from CAU; and 9 (16.1%) were seropositive for *Mycoplasma/Chlamydia pneumoniae*. The initial symptom severity was moderate-to-severe (UAS7 quartiles 18-32) and comparable between *Herpesviridae*-seropositive and *Herpesviridae*-seronegative patients. At 1, 6, and 12 months, UAS7 was consistently higher in seropositive children. In a multivariable analysis (adjusted for age, baseline UAS7, ASST, mean platelet volume, and other serology), *Herpesviridae* seropositivity was associated with higher UAS scores: mean difference 4.2 score points (95% confidence interval 0.5-7.9; Bayes estimate 4.2, 95% credible interval 1.2-7.3) in a mixed model for repeated measures. This estimate was comparable between children with positive (CAU) and negative (CSU) ASST.

**Conclusion:**

A history of CMV/EBV/HHV-6 infection might contribute to a slower-resolving CSU in children.

Chronic spontaneous urticaria (CSU) is a mast cell-driven disease characterized by recurrent wheals, angioedema, or both, persisting for most days of the week, for six weeks or longer (1). Chronic urticaria may be as frequent in children as in adults, equally affecting both sexes. The disease has a point prevalence of 0.5%-1.5% (2). When chronic urticaria is autoimmune in etiology, it is referred to as chronic autoimmune urticaria (CAU), which is characterized by degranulation of mast cells and basophils (FcϵRI) triggered by IgG against the high-affinity IgE receptor. CAU is often diagnosed *in vivo* with autologous serum skin test (ASST), although this test is characterized as a nonspecific screening test (3). Patients with a positive ASST present with severe clinical features and are more likely to have an accompanying autoimmune condition (4). A triggering/facilitating or predisposing factor for CSU is considered (albeit not without controversies) to be a bacterial, viral, or parasitical infection (5). Suggested mechanisms include the crosslinking between Toll-like receptors and FcϵRI on mast-cell surface by infectious agents, or direct degranulation by certain pathogens (6). A mild underlying infection or a pro-inflammatory state common in CSU can activate mast cells via neuropeptides, antimicrobial host defense proteins, or pro-inflammatory cytokines (7). In susceptible adults, infection-associated autoreactive immune response may induce chronic urticaria, at least the form with a positive ASST (7). Furthermore, reactivation of human herpes virus 6 (HHV-6) and/or of EBV infection was shown to be an important factor in the recurrence of CSU flares (8). In children, *Herpesviridae* infection is strongly associated with the onset of acute urticaria (9), but associations between specific viral pathogens and CSU have been scarcely investigated (10,11). We aimed to evaluate the relationship between serological signs of *Herpesviridae* infections (not necessarily acute) and the severity of symptoms in children with CSU over one year.

## Patients and methods

### Study outline

**Figure 1:**
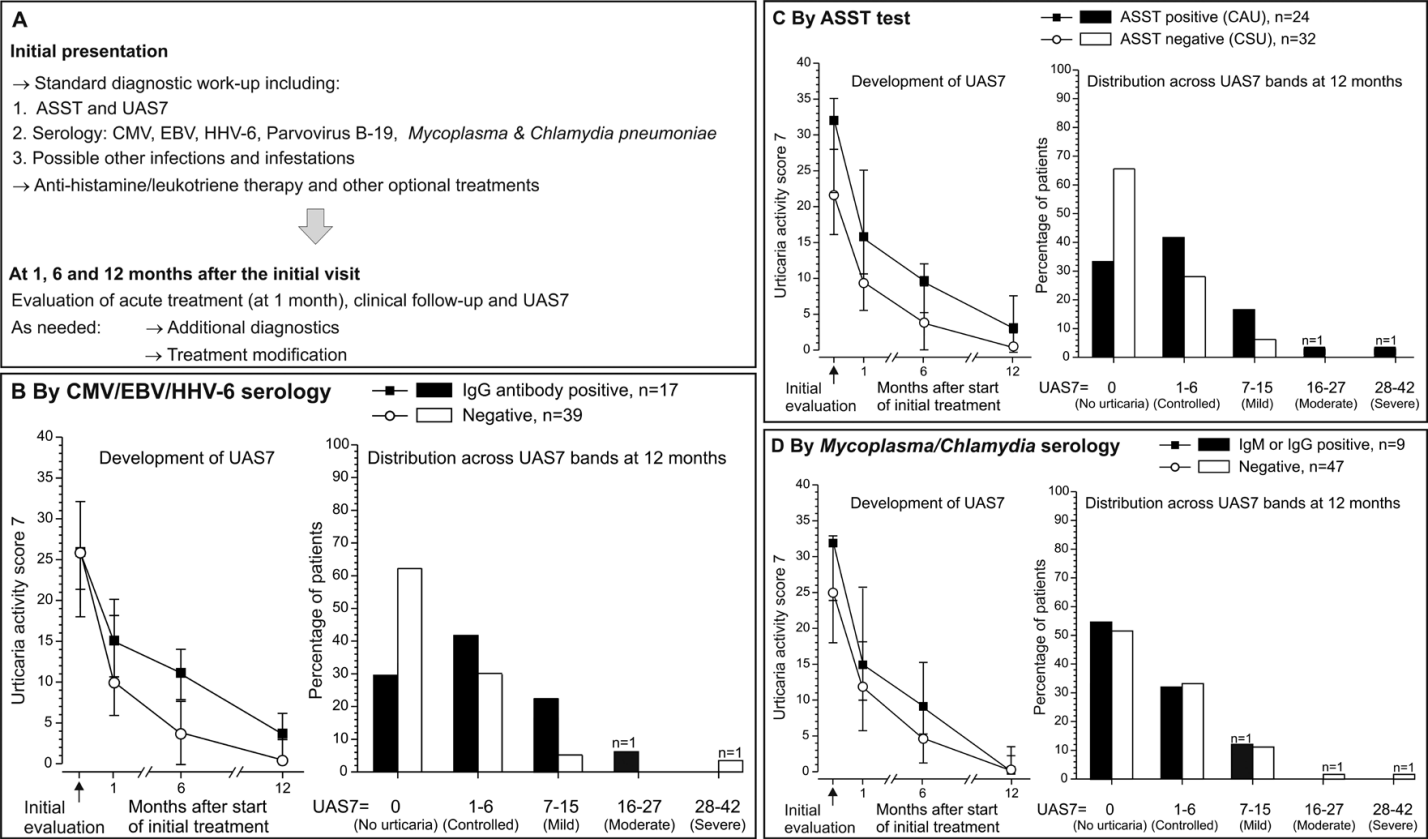
(**A)** Study outline. (**B-D**). Urticaria activity scores (UAS7) over time and distribution of patients across bands (12) of UAS7 at 12 months after initial treatment in subsets of patients based on the presence of IgG against cytomegalovirus (CMV), Epstein-Barr virus (EBV), human herpesvirus-6 (HHV-6); autologous serum skin test (ASST) result (chronic autoimmune urticaria [CAU] or chronic spontaneous urticaria [CSU]), and of antibodies against *Mycoplasma pneumoniae* or *Chlamydia pneumoniae*. Points are medians, bars represent lower and upper quartile, and columns depict percentages.

This prospective observational study was conducted at the University Hospital Center Zagreb, Croatia during 2016 and 2017. Consecutive children (age ≤18 years) with a new-onset flare of CSU underwent a standard diagnostic work-up (1), including the assessment of disease severity (urticaria activity score [UAS]) (12), the ASST, and a series of serological tests (Figure 1A). All patients received an initial antihistamine and/or antileukotriene treatment and sporadically short-term corticosteroids. Antibiotics were commenced in the cases of co-existing infections. The first re-evaluation followed after one month of the initial acute treatment. The patients were subsequently regularly evaluated at different time-intervals, but all had scheduled visits at 6 and 12 months after the initial evaluation (Figure 1A). Further clinical and laboratory evaluation was guided by patient-specific characteristics in respect to potential concomitant infectious diseases, allergies, or other possible conditions over time. The study was approved by the Ethics Committee of the University Hospital Center Zagreb.

### Patients

Children were included if (i) parents/guardians provided signed informed consent for the use of anonymized data collected through routine procedures for research purposes; (ii) CSU was diagnosed in line with the European Academy of Allergy and Clinical Immunology (EAACI) criteria (1); (iii) patients presented with a first disease manifestation or a new flare at least three months since the resolution of a previous one, with more than three months since the last corticosteroid and more than seven days since the last antihistamine dose. Children suffering from malignancy, immunodeficiency, diabetes mellitus, cardiovascular diseases, chronic renal failure, and systemic autoimmune diseases or using antithrombotic medication were not included.

### Patient management

The entire diagnostic work-up, treatment, and follow-up for all patients was managed by the same investigator (AČ) in line with the EAACI guidelines (1). ASST was performed within two days since the initial presentation while the urticarial rash was active and before the beginning of antihistamine/antileukotriene treatment. A serum-induced wheal ≥1.5 mm in diameter as compared with a saline-induced wheal at 30 minutes post-injection was considered a positive test result (13). Urticaria activity score (UAS) is a diary-based method that has been validated in adults, showing excellent clinimetric properties (12,14). UAS quantifies the disease severity (itch, hives) for a seven-day period (UAS7) (12). We applied once-daily UAS (itch and hives were assessed every 24 hours, in the evening) during seven consecutive days to create the UAS7, and all parents/guardians were trained to complete the diary. For every 24-hour period, UAS assigns between 0 and 3 points for itch (no itch to intense itch that interferes with daily activity or sleep) and for hives (no hives to >50 hives over 24 hours), for a maximum score of 42 (very severe urticaria) and a minimum of 0 (no urticaria). Symptoms diaries were kept continuously, and based on these records, UAS7 was calculated by the investigator at scheduled visits. At the initial presentation, where possible (milder disease form, not substantially interfering with normal daily activities or sleep), the standard antihistamine/antileukotriene treatment was postponed in order to obtain UAS7, otherwise it was started as soon as ASST was completed. Stool samples were evaluated for *C. albicans* colonization and parasitical infestation.

### Serological testing

Standard serological diagnostics were performed for EBV, CMV, HHV-6, and parvovirus B19 (1,4) as well as for *Mycoplasma pneumoniae* (15) and *Chlamydia pneumoniae* (16). IgM and IgG antibodies against EBV viral capsid (VCA), and early (EA) and nuclear (EBNA) antigens, as well as those against CMV and parvovirus B19, were detected with chemiluminescence assays. EBV reactivation was assumed based on the positivity for anti-EA EBV IgG with concomitant IgG antibodies to EBNA and VCA. Positive IgG anti-VCA and anti-EBNA were interpreted as a past EBV infection. Anti-*M. pneumoniae* IgM, IgG, and IgA were detected with an enzyme immunoassay, and anti-*C. pneumoniae* IgM, IgG, and IgA antibodies were detected with a microimmunofluorescence assay. According to the clinical presentation, positive IgM and/or IgA antibodies were considered a possible confirmation of an acute infection. Previous infections were defined according to IgG antibody presence.

### Outcomes

We used the UAS7 score as a measure of disease severity. Since in some patients, the initial score was obtained without the interference of treatment and in others it was potentially modified by an earlier treatment commencement, the primary outcome was the UAS7 score at 1, 6, and 12 months since the commencement of the initial anti-urticaria treatment. The secondary outcome was UAS7 score over the entire observation period (including the initial value) integrated as an area under the curve (AUC) (score*time).

### Statistical analysis

The primary outcome (longitudinal data) was analyzed by fitting generalized linear mixed models (hierarchical) to measured scores. The intention was to evaluate the association of serological indicators of *Herpesviridae* infections with the outcome. Pre-planned adjustments were age, initial UAS7, type of CSU (ie positive or negative ASST), visit (ie, time), and mean platelet volume determined at the initial work-up (as it seems to be associated with the severity of urticaria) (17). Other factors *a priori* considered as possible adjustments were other potential infections (if present). The secondary outcome, ie, AUC of the UAS7 score over time was ln-transformed and analyzed by fitting generalized linear models with the same effects as the primary outcome (except for time and the initial UAS7). Effects are expressed as geometric means ratios (GMR). In both cases, we used adaptive Gauss-Hermite quadrature estimation. To reduce the risk of “false findings” for both outcomes, estimates, estimated covariance matrices, and degrees of freedom were retained and used to adjust the observed confidence intervals and *P*-values of the differences between seropositive and seronegative children for multiplicity (logical-stepdown simulation method) (18). We report also (multiplicity adjusted) differences between children with a positive and a negative ASST, since ASST positivity is the best documented factor associated with more severe symptoms. Both outcomes were re-analyzed by fitting Bayesian models with the same effects. We used non-informative improper flat priors for fixed effects, and inverse gamma (shape = 2, scale = 2) for the scale and random variable (in the mixed model), conjugate sampling algorithm, burn-in size 2000, simulation size 50000, thinning 1. The analysis was conducted with SAS 9.4 for Windows software (SAS Inc., Cary, NC, USA).

## Results

None of the 56 included children had serological signs of an acute CMV, EBV, HHV-6, or parvovirus B19 infection (Table 1). Two children suffered from respiratory tract infections (Table 1): one tested positive for *M. pneumoniae* IgM and one for the common cold. One additional child tested positive for *M. pnemoniae* IgM and two tested positive for *C. pneumonie* IgM (all were asymptomatic) (Table 1). Two children, both asymptomatic, had a nasopharyngeal swab positive for group A Streptococcus. Stool colonization with *C. albicans* was common (41.1%) (Table 1). A negative ASST (CSU) was found in 32 children, and a positive ASST (CAU) was found in 24 children. (Table 1). Only 10 (17.8%) suffered from allergies, and 8 of 56 patients had a positive ASST (Table 1). Anti-thyroid peroxidase antibodies were found in two (no hormonal imbalances; anti-thyroglobulin antibodies negative in all children), hypogammaglobulinemia in three, and vitiligo in one child (Table 1). One child was subsequently diagnosed with mesenteric lymphadenitis of unknown cause, which eventually resolved. Overall, initial urticaria severity was predominantly moderate to severe (UAS7 quartiles 18-32) (Table 1). Expectedly, symptoms were more prominent in children with CAU (Table 1). Erythrocyte sedimentation rate and C-reactive protein levels (except for the child with an on-going respiratory tract infection), differential blood cell counts, total IgA, IgE, IgM, IgG, D-dimer levels, and complement C3 and C4 levels were all unremarkable (not shown).

**Table 1:** Patient characteristics based on the initial evaluation, overall and by autologous serum skin test (ASST) results: negative (chronic spontaneous urticaria) or positive (chronic autoimmune urticaria). Data are presented as count (percent) or median (quartiles, range)

	All children	Chronic spontaneous urticaria	Chronic autoimmune urticaria
N	56	32	24
Age (years)	10 (4-14.5; 1-18)	9.7 (3.3-13.8; 1-17)	10.2 (5-15; 1-18)
Boys	23 (41.1)	16 (50.0)	7 (29.2)
Initial urticaria activity score	26 (18-32; 10-40)	21.5 (16-28; 10-32)	32 (22-35; 12-40)
Allergic rhinitis, asthma, hypersensitivity	10 (17.8)	2 (6.2)	8 (33.3)
Clinically manifest respiratory infection	2 (3.6)	1 (3.1)	1 (4.2)
Anti-TPO antibodies	2 (3.6)	1 (3.1)	1 (4.2)
Hypogammaglobulinemia	3 (5.4)	2 (6.2)	1 (4.2)
Vitiligo	1 (1.8)	1 (3.1)	0
*Candida albicans* stool colonization	23 (41.1)	14 (43.7)	9 (37.5)
Parasitic infestation	0	0	0
CMV IgM positive	0	0	0
CMV IgG positive	4	3 (9.4)	1 (4.2)
Parvovirus B19 IgM positive	0	0	0
Parvovirus B19 IgG positive	5 (8.9)	4 (12.5)	1 (4.2)
HHV-6 IgM positive	0	0	0
HHV-6 IgG positive	8 (14.3)	6 (18.8)	2 (8.4)
EBV VCA IgM positive	3 (5.4)	0	3 (12.6)
EBV VCA IgG positive	10 (17.9)	5 (15.6)	5 (21.0)
EBV nuclear antigen IgM positive	0	0	0
EBV nuclear antigen IgG positive	10 (17.9)	5 (15.6)	5 (21.0)
EBV EA IgM positive	0	0	0
EBV EA IgG positive	0	0	0
*Mycoplasma pneumoniae* IgM positive	2 (3.6)	1 (3.1)	1 (4.2)
*Mycoplasma pneumoniae* IgG positive	6 (10.7)	4 (12.5)	2 (8.4)
*Mycoplasma pneumoniae* IgA positive	0	0	0
*Chlamydia pneumoniae* IgM positive	2 (3.6)	0	2 (8.4)
*Chlamydia pneumoniae* IgG positive	3 (5.4)	1 (3.1)	2 (8.4)
*Chlamydia pneumoniae* IgA positive	0	0	0
Mean platelet volume (fL)	10.7 (9.9-11.8; 7.0-11.9)	11.6 (9.9-11.9; 7.0-11.9)	10.5 (9.9-11.9; 7.0-11.9)

*Abbreviations: CMV – cytomegalovirus; EBV – Epstein-Barr virus; VCA – viral capsid antigen; EA – early antigen; HHV-6 – human herpesvirus-6, TPO – thyroid peroxidase.

Overall, 17 children (30.3%) tested positive for IgG antibodies against CMV, EBV, or HHV-6, and five of them tested positive for anti-parvovirus B19 IgG as well (Table 2). Seropositive children were somewhat older than the seronegative ones (Table 2), and were also somewhat more commonly seropositive for anti-*Mycoplasma/Chlamydia* antibodies (overall, nine children were positive for either IgM or IgG against these pathogens). However, seropositive and seronegative children had similar initial UAS7 scores, and a similar prevalence of positive ASSTs (Table 2).

**Table 2:** Patient characteristics by the presence of IgG against (cumulatively) cytomegalovirus, Epstein-Barr virus, and human herpersvirus-6. Data are presented as counts (percent) or median (quartiles, minimum-maximum)

	Seropositive	Seronegative
N	17	39
Age (years)	13 (9.3-15; 2.7-18)	7.8 (2.6-12.9; 1-17)
Boys	6 (35.3)	17 (43.6)
Initial urticaria activity score 7	26 (21.5-32; 12-34)	26 (18-32; 10-40)
Autologous serum skin test negative	10 (58.8)	22 (56.4)
Autologous serum skin test positive	7 (41.2)	17 (43.6)
*Mycoplasma/Chlamydia* antibody positive	6 (35.3)	3 (7.7)
*Candida albicans* stool colonization	8 (47.1)	15 (38.5)
Parvovirus B19 positive	5 (29.4)	0
Mean platelet volume (fL)	10.7 (9.9-11.9; 8.3-11.9)	10.7 (9.9-11.8; 7.0-11.9)

All patients started antihistamine/leukotriene treatment (Table 1). The one symptomatic child positive for anti-*M. pneumoniae* IgM antibodies was additionally treated with azithromycin, with a considerable decrease in UAS7 after one month (from 28 to 8 points). Of the two most severely affected children (both CAU), one was eventually treated with oral methylprednisolone over 24 days (tapering from 64 mg/day to 8 mg/day), and both suffered from an active disease at 12 months despite treatment (UAS7 26 and 32, respectively).

Although the initial UAS7 was practically identical in children who were positive and negative for anti-*Herpesviridae* IgG, over time it was consistently higher in the former group (Figure 1B). At the final assessment, more seropositive than seronegative children had some urticaria activity (Figure 1B). In children with CAU, UAS7 was consistently higher at all evaluations than in children with CSU (Figure 1C). At the final assessment, most children with CSU were disease-free, while most of the children with CAU had some disease activity still present (Figure 1C). In children with antibodies against *Mycoplasma/Chlamydia,* the initial UAS7 was markedly higher than in children without them, but this difference diminished over time (Figure 1D). UAS7 scores in children with or without *C. albicans* stool colonization were consistently similar (not shown).

In a multivariable analysis of the primary outcome, CMV/EBV/HHV-6 seropositivity (vs seronegativity) was independently associated with a higher UAS7 over time (Table 3). This was consistent in children with CAU (ie, positive ASST) and children with CSU (ie, negative ASST), although the present sample was too small for a meaningful test of effect modification (Table 3). CAU was also associated with higher UAS7 scores (compared with CSU) (Table 3). Similar results were observed in a multivariable analysis of the secondary outcome (area under the curve of UAS over time) (Table 3).

**Table 3:** Summary of a multivariable^†^ analysis of the primary outcome (urticaria activity score, UAS7, at visits 1, 6, and 12 months after commencement of initial treatment) and of secondary outcome (UAS7 over time integrated as area under the curve from the initial visit to month 12). Effects are mean differences (D) for the primary outcome and geometric means ratios (GMRs) for the secondary outcome. Shown are estimates from frequentist models with multiplicity unadjusted and adjusted confidence intervals (CI) and *P* values, and Bayes estimates with credible intervals (CrI) and probabilities (P) that D>0, ie, that GMR from >1.0

	Primary outcome		
	Multiplicity unadjusted	Multiplicity adjusted	Bayes

	D (95% CI)	P	95% CI	P	D (95% CrI)	P (%)
CMV/EBV/HHV6 IgG positive	4.2 (1.1, 7.2)	0.007	0.5, 7.9	0.021	4.2 (1.2, 7.3)	99.6
ASST positive (CAU)	3.3 (0.4, 6.2)	0.025	-0.2, 6.8	0.049	3.3 (0.3, 6.3)	99.4

	Secondary outcome					
	GMR (95% CI)	P	95% CI	P	GMR (95% CrI)	P (%)
CMV/EBV/HHV6 IgG positive	1.72 (1.20-2.45)	0.004	1.10-2.67	0.007	1.71 (1.13-2.52)	99.5
ASST positive (CAU)	2.04 (1.50-2.78)	<0.001	1.39-3.00	<0.001	2.04 (1.44-2.89)	100

*Abbreviations: ASST – autologous serum skin test; CAU – chronic autoimmune urticaria; CMV – cytomegalovirus; EBV – Epstein-Barr virus; HHV-6 – human herpesvirus-6.

†For the primary outcome, the model included additional fixed effects: age, initial UAS7, mean platelet volume, *Mycoplasma/Chlamydia* serology, and time. The same adjustments were made for the secondary outcome, except for the initial UAS7 and time. The cohort was too small for a meaningful test of interaction between the serological indicators of *Herpesviridae* contact and ASST result. However, in models re-fitted to include the interaction term, the differences between the *Herpesviridae*-seropositive and seronegative children were similar in those with a positive and those with a negative ASST: (i) for the primary outcome, mean difference = 3.1 (95% CI -1.4, 7.6) (Bayes estimate = 3.1, 95% CrI -0.72, 6.9) in children with a negative ASST; and mean difference = 5.9 (95% CI 0.4, 11.5) (Bayes estimate = 6.0, 95% CrI 1.3, 10.6) in children with a positive ASST; (ii) for the secondary outcome, GMR = 1.85 (95% CI 1.06-3.22) (Bayes estimate = 1.85, 95% CrI 1.12-3.10), in children with a negative ASST, and GMR = 1.54 (95% CI 0.79-3.00) (Bayes estimate = 1.54, 95% CrI 0.83-2.80), in children with a positive ASST.

## Discussion

In this study, the history of CMV/EBV/HHV-6 infection contributed to a slower-resolving CSU in children. EAACI guidelines (1) suggest the importance of infection in the etiology of CSU, hence the present microbiological and serological evaluations were conducted according to these guidelines (1). In adults with CSU, infection/reactivation of HHV-6 and of EBV infections triggers the recurrence of disease flares (8). In children with CSU, such evidence is missing (we repeated several Medline searches in 2022 and January 2023). Also, HSV-1, HSV-2 infections, which are considered a likely causative factor in isolated acute urticaria episodes in children (10,19), are not considered related to pediatric CSU (11). Moreover, seronegativity to HSV-1 and HSV-2 was found to be associated with recurrent acute episodes (19). Thus far, more severe symptoms at presentation (higher UAS) and positive ASST appear to be the only indicators associated with slower symptom resolution in children with CSU (20,21).

In the present study, we investigated whether *Herpesviridae* infection, which has been implicated in CSU course in adults (8), was also related to disease severity/resolution in children with CSU. In this respect, the study is limited by the fact that it relied upon standard routine procedures and only clinically indicated evaluations were undertaken – children were not systematically re-assessed for *Herpesviridae* serology, and no direct viral diagnostic procedures were undertaken. Hence, the study could not have captured potential viral reactivation or new-onset infections over time. At baseline, none of the included patients suffered from acute *Herpesviridae* infection. However, 17/56 (30.4%) had IgG antibodies against (cumulatively) CMV/EBV/HHV-6 viruses (five of them were also positive for past contacts with parvovirus B19) (seropositive). While seropositive and seronegative patients presented with virtually identical UAS scores, seropositive patients showed consistently higher scores at 1, 6, and 12 months after the start of treatment. Furthermore, with adjustment for age, baseline score, ASST result, *Mycoplasma/Chlamydia* serology, and mean platelet volume, seropositive patients had clearly higher time-averaged UAS scores and the area under the symptom score. Moreover, this difference was consistent in children with a negative and children with a positive ASST (CAU). Due to the mentioned limitations, the reported estimates might have been biased by unrecorded viral reactivation in seropositive children or by an unrecorded new-onset infection in children initially classified as seronegative. However, even if such events did occur, they would not have materially changed the conclusion about the association between serological indicators of a past contact with *Herpesviridae* and a slower disease resolution. Based on clinical development in individual children, it seems highly unlikely that any of those potential events indeed happened – all but two children (both with CAU) reacted well to the initial antihistamine/antileukotriene treatment, and average and individual symptom severity gradually subsided.

As an additional finding, children with a positive ASST presented with more severe symptoms than children with a negative ASST, and the difference persisted over the observed 12 months. The association also remained apparent in multivariable models. This finding is in line with the observation that ASST-positive patients generally present with more severe/persistent symptoms (4,22). It is possible that the children classified as ASST-positive differed among themselves in the intensity of the immune response, in particular, the two children with the most severe and persistent symptoms – potentially discernible by the basophil activation test (BAT) (22). In a recent study in adults with CSU, UAS7 was highest in ASST-positive patients with a positive BAT, lower in ASST-positive patients with a negative BAT, and lowest in ASST-negative patients with a negative BAT (22). In the mentioned study, none of the 139 evaluated patients had a negative ASST and a positive BAT (22). Therefore, BAT is complementary to ASST and might serve as a laboratory marker of more severely affected patients (22). However, it is not routinely undertaken in daily practice (22), and was not applied in the present cohort. We do not consider this fact to be relevant with respect to our primary objective. An insight into the BAT results would not have had any repercussion on the present estimates of differences between *Herpesviridae*-seropositive and seronegative children, since we adjusted for the ASST status (and several other potential confounders).

A number of molecular mechanisms might link infections to CSU (7). Overall, however, it is likely that infection acts as a facilitating factor for the initiation and perpetuation of CSU, but additional cofactors seem to be required for the CSU phenotype to be expressed (7). The present study was not a mechanistic one and makes no contribution in this respect. Nevertheless, the present findings are in line with observations in adults with CSU, where EBV/HHV-6 infection/reactivation was suggested as a likely cause of recurrence of the disease flares (8).

In conclusion, in the present cohort of children with CSU, those who at baseline had serological evidence of a past contact with *Herpesviridae* (specifically CMV/EBV/HHV-6) presented with more severe symptoms than their seronegative counterparts. However, based on repeated assessment at 1, 6, and 12 months after the start of treatment, and accounting for several potential confounders, symptom severity was consistently higher in seropositive than in seronegative children, which suggests slower symptom resolution. The present observations agree with observations linking HHV-6/EBV infection with disease flares in adults with CSU.
